# Electroencephalographic Fractal Dimension in Healthy Ageing and Alzheimer’s Disease

**DOI:** 10.1371/journal.pone.0149587

**Published:** 2016-02-12

**Authors:** Fenne Margreeth Smits, Camillo Porcaro, Carlo Cottone, Andrea Cancelli, Paolo Maria Rossini, Franca Tecchio

**Affiliations:** 1 LET’S—ISTC—CNR, Ospedale Fatebenefratelli, Isola Tiberina, Rome, Italy; 2 University of Amsterdam, Amsterdam, The Netherlands; 3 Institute of Neuroscience, Newcastle University, Medical School, Newcastle upon Tyne, United Kingdom; 4 Institute of Neurology, Cattolica del Sacro Cuore University, Rome, Italy; 5 Unit of Neuroimaging, IRCCS San Raffaele Pisana, Rome, Italy; 6 Department of Information Engineering, Università Politecnica delle Marche, Ancona, Italy; & National Laboratory of Pattern Recognition, CHINA

## Abstract

Brain activity is complex; a reflection of its structural and functional organization. Among other measures of complexity, the fractal dimension is emerging as being sensitive to neuronal damage secondary to neurological and psychiatric diseases. Here, we calculated Higuchi’s fractal dimension (HFD) in resting-state eyes-closed electroencephalography (EEG) recordings from 41 healthy controls (age: 20–89 years) and 67 Alzheimer’s Disease (AD) patients (age: 50–88 years), to investigate whether HFD is sensitive to brain activity changes typical in healthy aging and in AD. Additionally, we considered whether AD-accelerating effects of the copper fraction not bound to ceruloplasmin (also called “free” copper) are reflected in HFD fluctuations. The HFD measure showed an inverted U-shaped relationship with age in healthy people (R^2^ = .575, *p* < .001). Onset of HFD decline appeared around the age of 60, and was most evident in central-parietal regions. In this region, HFD decreased with aging stronger in the right than in the left hemisphere (*p* = .006). AD patients demonstrated reduced HFD compared to age- and education-matched healthy controls, especially in temporal-occipital regions. This was associated with decreasing cognitive status as assessed by mini-mental state examination, and with higher levels of non-ceruloplasmin copper. Taken together, our findings show that resting-state EEG complexity increases from youth to maturity and declines in healthy, aging individuals. In AD, brain activity complexity is further reduced in correlation with cognitive impairment. In addition, elevated levels of non-ceruloplasmin copper appear to accelerate the reduction of neural activity complexity. Overall, HDF appears to be a proper indicator for monitoring EEG-derived brain activity complexity in healthy and pathological aging.

## Introduction

Various forms of dementia, particularly Alzheimer’s Disease (AD), are among the most common disorders in the elderly population [1]. A deeper understanding of the mechanisms involved in the development and progression of AD is critical for early diagnosis and effective treatment. The characteristic structural neuropathology of AD involves neuronal cell loss, neurofibrillary tangles and amyloid plaques [2]. On the functional neural level, profound changes in brain activity can be observed in AD patients. Specifically, AD patients display a ‘slowing’ of brain activity, marked by an increase of electroencephalographic (EEG) power in low-frequency rhythms, including delta (0–4 Hz), theta (4–8 Hz) and low alpha (8–10 Hz) bands, and a decrease in EEG power and cortico-cortical coherence in higher-frequency brain rhythms (high alpha (10–13 Hz) and beta (13–33 Hz) bands) [1]. Slowing of brain activity is not selectively present in cognitive impaired or AD patients, but to a milder degree also occurs during healthy aging [3–5].

### Electroencephalographic Fractal Dimension

Measuring complexity of brain activity is proposed as a useful indicator of (early) AD diagnosis and assessment of neuropsychological functioning in AD patients [1,6,7]. A network system is said to have high complexity if it is both highly specialized and highly integrated [8]. Complexity of EEG-derived brain activity mirrors the level of interaction between different elements of the neural system [9]. The level of interaction between different functional networks in the brain fluctuates over time in a characteristic way. These characteristic fluctuations of interactions cause a pattern in brain activity that is self-similar over different spatial and temporal scales, i.e. displays fractal properties [9]. In other words, neural activity shows similar features over and over again on a scale-free basis [9,10]. This type of complex structure in brain activity can be quantified by the fractal dimension [9].

Loss of complexity in brain activity means that the neural system of the brain is less flexible and less efficient in processing [11–13]. AD patients show a decline in complexity of neural activity over the whole brain, demonstrated by reductions in fractal dimension or other complexity measures of EEG time series [6,7,14], with further complexity decline related to progressing cognitive impairment [1,15]. Also, healthy aging individuals show decreasing complexity in brain activity and in synchronization among the oscillating rhythms produced by different neural areas, as shown by several measures of complexity [13,16]. However, to date, it remains unclear whether or not the EEG-derived fractal dimension measure specifically, as it is sensitive to neural changes typical of AD, is also sensitive to neural changes related to healthy aging.

### Homologous Areas Interhemispheric Symmetry

In addition to the degree to which brain activity demonstrates complexity, differences in complexity of brain activity between homologous areas in the left and right hemisphere may provide insight into cognitive and neural changes related to healthy aging and AD. A number of studies have shown that a decline in the symmetry between homologous brain activities and brain structures is associated with reduced working efficiency of the brain in healthy aging and in AD disease progress [17–19]. Moreover, it has been shown that interhemispheric asymmetry in EEG-derived fractal dimension is sensitive to cognitive states. For example, EEG-derived fractal dimension asymmetry is associated with a more severe experience of negative emotions in patients with a history of depression [20], predicted a worse function outcome in stroke patients [13], and seems to be typical of waking and sleep stages I and II as opposed to deep-sleep stages III and IV [21].

Altogether, these studies suggest that homologous areas interhemispheric symmetry of the fractal dimension measure may provide additional information about cognitive and neural changes in healthy aging and AD.

### Alzheimer’s Disease and Copper Metabolism

AD severity and progression are hypothesized to be related–together with several other risk factors–to increased levels of copper which is not structurally bound to ceruloplasmin (non-Cp copper (NCC), also called ‘free’ copper, or labile copper) in a subgroup of susceptible individuals [22]. Pal and colleagues [22] found that elevated NCC levels in AD patients are associated with a higher conversion rate from Mild Cognitive Impairment (MCI) to AD, faster disease progression in AD patients, and cognitive decline among both AD and healthy subjects. NCC can cross the blood-brain barrier and damages the brain in two ways: it leads to increased oxidative stress in neural cells and contributes to the development of amyloid plaques [22]. Cognition and neural function is found to be affected by increased levels of NCC in the AD and in the healthy brain [23,24]. Similar relationships may be expected with the fractal dimension measure, since complexity and a self-organizing structure of brain activity is associated with better neuronal functionality [12,25].

### Aim

The aim of the present study was to investigate whether the fractal dimension is sensitive to decreases in neural efficiency typical of healthy and/or pathological brain aging. Accordingly, we expect that in healthy individuals the fractal dimension will change with age. Furthermore, we aim to replicate results showing that the fractal dimension is sensitive to alterations in the functional organization of the brain in AD patients, mirroring a further reduction of brain activity complexity relative to healthy elderly individuals. In addition, we hypothesize that reductions in fractal dimension are associated with more severe cognitive impairment. Finally, based on the neurodegenerative decline secondary to high NCC levels in AD patients, we hypothesize that higher NCC levels correspond to reduced brain activity complexity levels. Specifically, while the main interest of other studies [6,7,14] has been AD diagnosis, here our focus was on the regional specificities of complexity alterations in AD with respect to healthy aging, on the inter-hemispheric asymmetry of homologous areas’ complexities, and on variations in such indices according to NCC levels. In fact, we think that a deepened understanding of AD-correlates of brain networks’ fundamental properties (like regional specific brain activity complexity) may enhance the identification of individual readout measures for specific therapeutic interventions.

For these purposes we estimated Higuchi’s fractal dimension (HFD) [11] of EEG-derived brain activity from AD patients and from healthy individuals with ages from across the life-span, starting from late adolescence. We studied HFD dependence on age in healthy people as well as on cognitive level and NCC in AD, with attention to possible regional prevalence of the observed effects. We considered HFD instead of other complexity measures for two reasons. First, we would like to consider mathematical tools that go beyond the classic Fourier analysis (Fast Fourier Transform, FFT). In fact, FFT works upon the hypothesis of stationary signals. Instead, we now know that even the resting state exhibits dynamics; the network transits over time through multiple activated-deactivated states resulting in a non-stationary regime [26–29]. Proceeding in this direction, we noticed that the fluctuation frequencies of the population’s neuronal activity display the so-called ‘power law’ dependence [30–32]. Since the signal fractal dimension simply corresponds to the exponent of this exponential function, we considered the fractal dimension a good candidate for measuring brain activity complexity. Second, HFD was the single best performer in a study comparing the discriminating ability of diverse measures between healthy elderly and those affected by AD [6]. The authors compared HFD, spectral entropy (disorder in the spectrum), spectral centroid (a measure of spectral shape), spectral roll-off (the frequency sample below which a specific percent of the spectral magnitude distribution is contained), and zero-crossing rate (the rate at which the signal changes sign): HFD discriminated in about 67% of cases while all other indices were below 63%.

## Materials and Methods

The Ethics Committee of the ‘San Giovanni Calibita’ Hospital, Isola Tiberina, Rome approved this study’s protocol. All participants or legal guardians signed written informed consent.

### Participants

#### AD patients

We recruited 67 individuals with a diagnosis of probable AD according to NINCDS-ADRDA criteria [33] and a Mini-Mental State Examination score of 24 or less (MMSE) [34]. Benzodiazepines, antidepressants or antihypertensives were suspended 24 hours before the EEG recordings to make drug conditions comparable across subjects [23].

#### Healthy control subjects

We recruited 41 healthy subjects in the age range 18–85 years, with no signs of cognitive impairments or neurological or psychiatric history. We selected a subgroup of healthy people matching for age with the AD group (17 subjects, age range 51–85 years), to constitute elderly controls (EC) for the AD group. EC and AD groups did not differ for age and education (*t*(79) = -1.37, *p* = .175 and *t*(73) = 0.06 *p* = .952 respectively, Table 1). We called the remaining participants young controls (YC). Exclusion criteria were: symptoms of cognitive impairments (MMSE < 24), geriatric depression (Geriatric Depression Scale [35] < 14), and diagnosis of AD or other forms of dementia according to NINCDS-ADRDA and DSM IV criteria and neuroimaging diagnostic procedures using computer tomography (CT) or magnetic resonance imaging (MRI).

**Table 1:** Descriptive statistics.

	Gender (M/F)	Age	Years of education	MMSE	Average HFD
**Young Controls**	10/14	30.3 ±7.52	-	-	1.943 ±0.010
**Elderly Controls**	6/11	69.5 ±9.46	9.27 ±4.45	27.6 ±1.60	1.917 ±0.021
**Alzheimer Patients**	21/46	73.0 ±8.58	9.18 ±4.81	20.6 ±4.25	1.895 ±0.042

Descriptive statistics represented as number of participants per category or mean ±SD. HFD is averaged across the whole brain and over subjects per group.

### Non-Ceruloplasmin-Bound Copper Levels

Blood samples were collected from EC and AD subjects during a morning visit in fasting conditions. Blood serum was stored at −80°C until biochemical measurements of NCC were performed. For each serum copper and ceruloplasmin pair, we computed the amount of copper bound to ceruloplasmin (CB) and the amount of NCC, following standard procedures [36], as:

NCC=Absolute serum copper-CB

CB = ceruloplasmin (mg/dL) * 10* n, where n = 0.0472 (μmol/mg).

NCC concentration, absolute serum copper concentration and ceruloplasmin concentration are all represented in μmol/L. This calculation is based on the fact that ceruloplasmin binds 6 atoms of structural copper, which is equivalent to an atom weight percentage of copper of 0.3% in ceruloplasmin.

(more details are available at http://www.jalz.com/letterseditor/index.html#March2013).

### Electroencephalography (EEG)

Resting-state 19-channels EEG were recorded for a duration of 5 minutes while participants were awake with their eyes closed and sitting in a comfortable chair. Electrodes were positioned according to the 10–20 international system (Fp1, Fp2, F7, F3, Fz, F4, F8, T3, C3, Cz, C4, T4, P3, Pz, P4, P7, P8, O1, O2) and a fronto-central reference was used. EEG data were filtered between 0.1–100 Hz, sampled at 128 Hz and stored for offline analysis.

First, the data were reviewed, and an EEG expert manually discarded the epochs with artefactual waveforms. Second, the detection and rejection of artifacts were completed through independent component analysis (ICA) using a semi-automatic ICA procedure [37]. ICA is a blind source decomposition algorithm that enables the separation of statistically independent sources from multichannel data. It has been proposed as an effective method for separating biological (ocular, cardiac and motor artifacts) and non-biological artifacts (Power line, environmental noise) from EEG data [38–40]. The components were visually inspected. If artifact contaminations were found, the investigator manually rejected them. Third, we down-sampled all the EEG time series to a signal with a sampling frequency of 128 Hz, according to the lowest sampling frequency present in the EEG data in hand. Finally, the data were re-referenced to an average reference.

**Fig 1:**
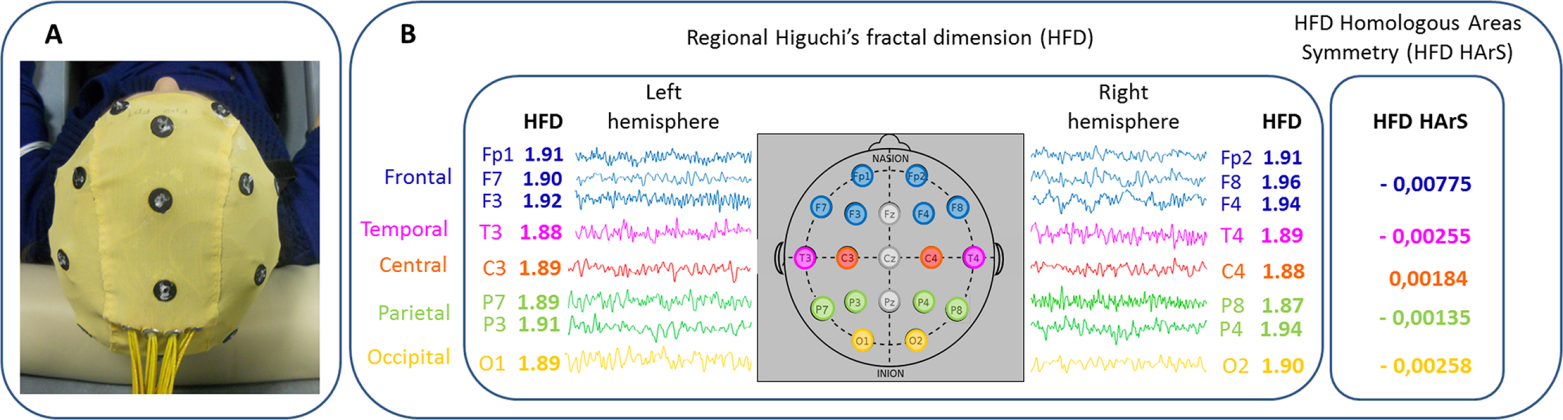
Experimental setup. A) 19-channel EEG cap. B) Regional HFD and HFD Homologous areas symmetry (HArS) estimates (explicative values). HFD is calculated over the whole time series measured at each derivation (windows of 2-seconds are displayed). HFD values are averaged across multiple derivations that overlay the same brain region (frontal and parietal). HArS estimates left vs. right normalized HFD difference (see methods).

We estimated Higuchi’s fractal dimension (HFD, see description below) directly from the EEG time series. We considered?? regional HFD by grouping the corresponding channels in each of the two hemispheres (frontal left: Fp1, F7, F3; frontal right: Fp2, F4, F8; central left: C3; central right: C4; temporal left: T5; temporal right: T4; parietal left: P3, P7; parietal right: P4, P8; occipital left: O1; occipital right: O2) (Fig 1). Subsequently, we calculated the HFD homologous areas interhemispheric symmetry (HArS) with a formula detailed below.

### Higuchi’s Fractal Dimension

Higuchi’s fractal dimension [11] of a time series is a number between 1 and 2, with higher HFD values corresponding to higher levels of signal complexity. A signal is fractal if the scaling properties fit a scale-free behavior, i.e. the same features emerge at smaller and larger time scales [9]. The EEG signal of brain activity shows fractal properties, since the dynamics of the EEG time series displays statistical similarities at different time scales [30–32].

According to the concept of quantifying the emergence of similar features at different time scales, Higuchi’s algorithm uses many time series built by down-sampling the original signal y(t) every k samples. From the original *N*-sample EEG time series y(t) [*y*(1), *y*(2), …, *y*(*N*)], Higuchi’s algorithm defines k_max_ (k_max_ >1) new time series:

$$y_{k}^{m}:y\left(m\right),y\left(m+k\right),y\left(m+\mathrm{2k}\right),\;\ldots ,y\left(m+\text{int}\;\left(\frac{N\;-\;m}{k}\right)k\right)$$

where *m* is the first sample and k goes from 1 to k_max_.

The length *L*_*m*_(*k*) of each curve $y_{k}^{m}$ is calculated as follows:

$$L_{m}\left(k\right)=\frac{1}{k}\left[\frac{N\;-\;1}{\text{int}\left(\frac{N\;-\;m}{k}\right)\;k}(\sum_{i=1}^{\text{int}\left(\frac{N\;-\;m}{k}\right)}|y\left(m\;+\;i\;k\right)\;-\;y\left(m\;+\;\left(i\;-\;1\right)k\right)|)\right]$$

The calculation of the curve length is repeated for k from 1 to *k*_max_.

For each possible k time step, the length of the curve L(k) is evaluated by averaging the k sets of *L*_*m*_(*k*) values, as in:

$$L(k)=\frac{1}{k}\sum_{m=1}^{\text{k}}\;L_{m}(k)$$

If L(k)∼k^−FD^, then the curve is fractal with dimension FD. In that case, the plot of log(L(k)) against log(1/k) should fall on a straight line with slope equal to -FD. Therefore, FD can be obtained by means of a least-squares linear best-fitting procedure.

**Fig 2:**
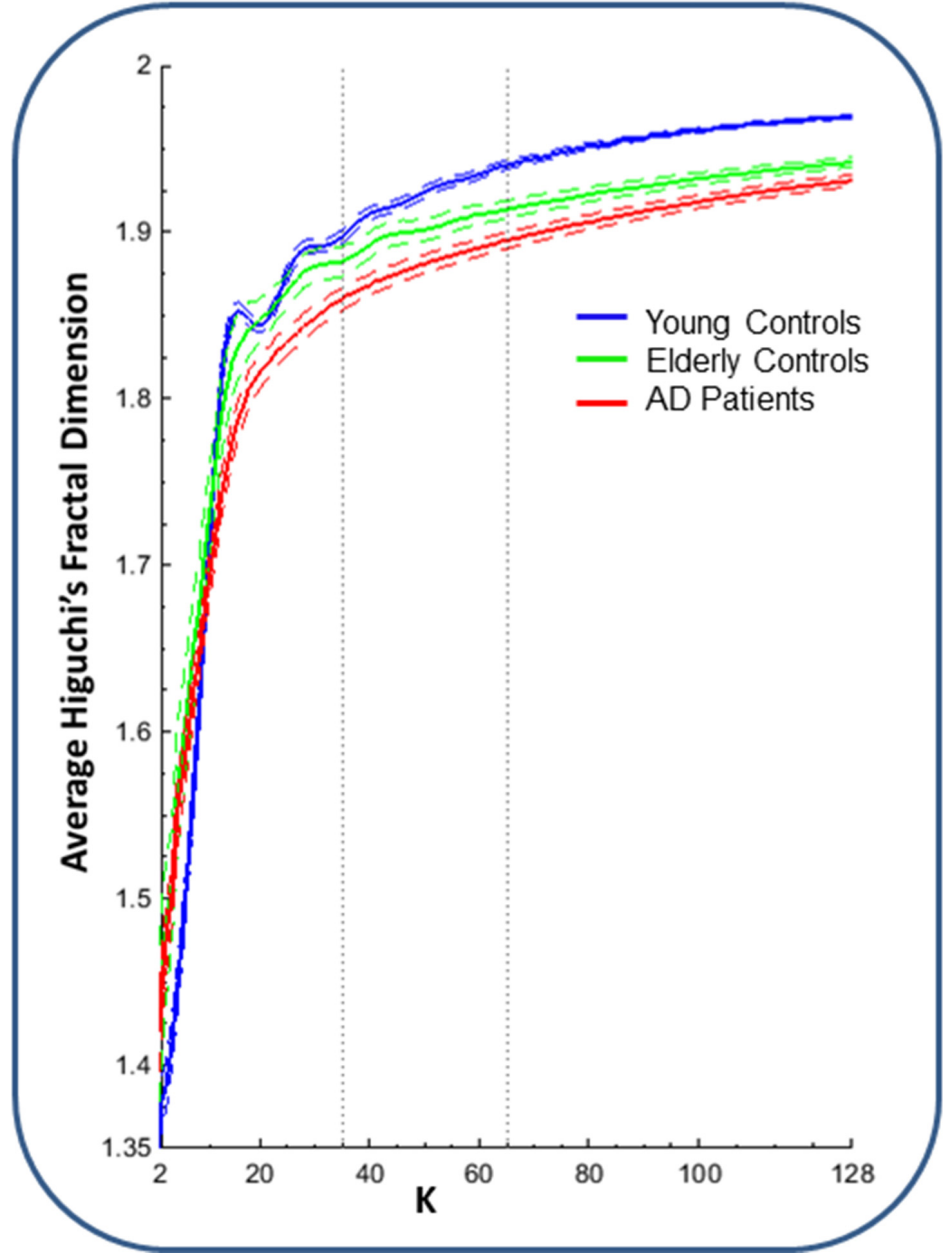
Whole-brain average HFD in dependence on *k*_*max*_. Dashed curves represent the standard error of the mean. Dotted lines at *k*_*max*_ = 35 and *k*_*max*_ = 65 represent, respectively, the starting point of HFD stability with respect to the difference between groups, and the selected *k*_*max*_ in our study.

Importantly, HFD does not depend on signal amplitude. For each channel, we calculated HFD in windows of 2 seconds (in our data: 256 samples) and averaged over time. The only parameter of Higuchi’s algorithm is *k*_*max*_. HFD increases with higher values of *k*_*max*_. We estimated HFD for *k*_*max*_ = 2, …, 128 –with a maximum of 128 because this corresponds to half of the 2-second windows (128 samples)–to examine the relationship between HFD values in our groups of interest (YC, EC and AD) with different values of *k*_*max*_. We observed that the relationship between the three groups remained stable starting from a *k*_*max*_ > 35 (Fig 2). However, we chose *k*_*max*_ = 65, as this value corresponds to the median whole-brain average HFD value of the entire sample obtained for *k*_*max*_ = 2, …, 128.

### Homologous Areas Interhemispheric Symmetry (HArS)

In addition to looking at HFD magnitude, we also investigated symmetry in HFD between homologous areas in the left and right hemisphere. To this end we calculated:

$$\text{HFD HArS}=\frac{\text{HFD Left Channel}\;-\;\text{HFD Right Channel}}{\text{HFD Left Channel}\;+\;\text{HFD Right Channel}}$$

According to this calculation, left-higher-than-right HFD asymmetries correspond to positive values. Instead, right-higher-than-left HFD asymmetries correspond to negative values. We averaged the HFD homologous asymmetry values for regions with more than one channel (see above, Fig 1).

### Statistical Analysis

In order to examine the relationship of neural/neural or neuronal? activity complexity with on one side, age in healthy people, and on the other side cognitive status and NCC in AD, correlations and best fit curve were estimated for the association between HFD and the corresponding variable. To evaluate whether possible relationships were more evident in specific brain regions, HFD was entered into an ANOVA model with *Channel Region* (Frontal, Central, Temporal, Parietal, Occipital) and *Channel Hemisphere* (Left, Right) as within-subject factors, and 3-level *Group* as a between-subjects factor. The *Group* factor included YC, EC and AD groups to study the effects of aging in healthy people and in AD patients together with the effects of AD. When the full ANOVA model displayed interaction effects, we performed reduced ANOVA models and we examined further the main effects by using Bonferroni-corrected post-hoc tests. We focused on post-hoc comparisons YC vs. EC and EC vs. AD, omitting the YC vs. AD comparison.

Group differences in HFD HArS were analysed using a one-way ANOVA model with *HFD HArS* per region (Frontal HArS, Central HArS, Temporal HArS, Parietal HArS and Occipital HArS) as the dependent variable, and *Group* (YC, EC, AD) as between-subject factor. We further examined the dependence of regional HFD HArS on aging and cognitive impairment by calculating the correlations with age and MMSE.

The results were analysed using SPSS 20.0 and reported only when significant. We considered *p* < .050 as the significance threshold and we report trends for effects with *p* < .100, with significance properly corrected for multiple comparisons (Bonferroni correction) and the violation of sphericity assumption (Greenhouse-Geisser correction).

## Results

### Participants

Since females tend to have a slightly higher HFD than males [41], we made sure the groups of our sample had similar gender ratios (no differences between YC and EC, nor between EC and AD, *t*(37) = -.51, *p* = .614 and *t*(79) = -.23, *p* = .821 respectively, Table 1). Evidently, the AD group showed significantly lower MMSE scores than the EC group (*t*(82) = 6.66, *p* < .001, Table 1).

### Correlations of Higuchi’s Fractal Dimension with Age and Cognitive Impairment

**Fig 3:**
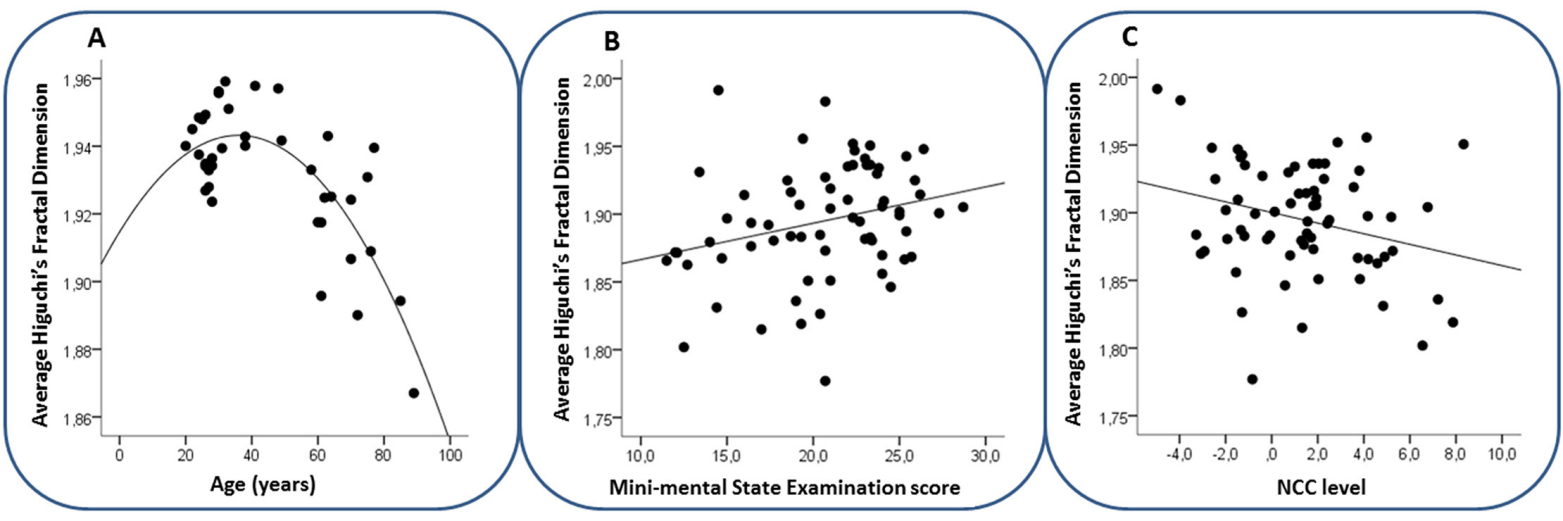
**Scatter plot of average whole-brain HFD values** of A) Healthy subjects in relationship with age (years), with the best fitting curve (R^2^ = .575, *p* < .001); B) AD patients with MMSE scores. We considered that MMSE values do not distribute as a Gaussian, reporting the Spearman’s correlation in the results. Nevertheless, for graphical representation we reported the line corresponding to the Pearson’s correlation (also below significance threshold r = .264, p = .031). C) AD patients with NCC levels, with best fitting line (*r* = -.274, *p* = .025).

To analyse the correlation between HFD and age, we used Pearson’s Rho as both variables’ distributions did not differ from a Gaussian. Among YC we found a trend of positive correlation between age and average HFD (*r* = .372, *p* = .074). In contrast, the EC group showed a significant negative correlation between age and average HFD (*r* = -.538, *p* = .038). Analysing all healthy subjects as a single group (YC and EC together), a parabola was the best fitting curve for HFD in relationship with age (Fig 3).

Because MMSE scores were non-normally distributed within the sample of AD patients (Shapiro-Wilks *W*(67) = .957, *p* = .021), we used Spearman’s Rho to analyse its correlation with HFD. We found a positive correlation between MMSE and average HFD among all elderly subjects (EC and AD together, *r* = .343, *p* = .001) as well as within the AD sample separately (*r* = .297, *p* = .015).

NCC levels correlated negatively with average whole-brain HFD (Pearson’s *r* = -.289, *p* = .008 including EC and AD, Fig 3 for AD group). This trend remained when controlling for MMSE scores (Pearson’s *r* = -.211, *p* = .057 among EC and AD, *r* = -.215, *p* = .084 in AD only).

To test whether the above-mentioned correlations had a regional-specific prevalence, we executed the full ANOVA model on HFD magnitude. In addition to the trends of group differences when comparing both YC vs. EC (*p* = .077) and EC vs. AD (*p* = .053), we observed a significant three-way interaction effect of Channel Region*Channel Hemisphere*Group (*F*(7.17,369.39) = 5.57, *p* < .001, Greenhouse-Geisser corrected). To examine this interaction, we tested reduced ANOVA models separated for *Channel Region* (Frontal, Central, Parietal, Occipital, Temporal), with *Channel Hemisphere* as within-subjects factor and *Group* as between-subjects factor. In the comparison of healthy groups, YC differed from EC in central (*p* = .002) and parietal (*p* = .032) regions. Instead, AD showed differences from EC in temporal (*p* = .016) and occipital (*p* = .038) regions.

To further test spatial-dependence of HFD differences, we checked whether NCC levels of AD-affected people correlated with brain complexity in a spatially-specific manner. We observed that the strongest correlation occurred between NCC and parietal HFD (Pearson’s *r* = -.341, *p* = .005).

### HFD Homologous Areas Interhemispheric Symmetry (HFD HArS)

**Fig 4:**
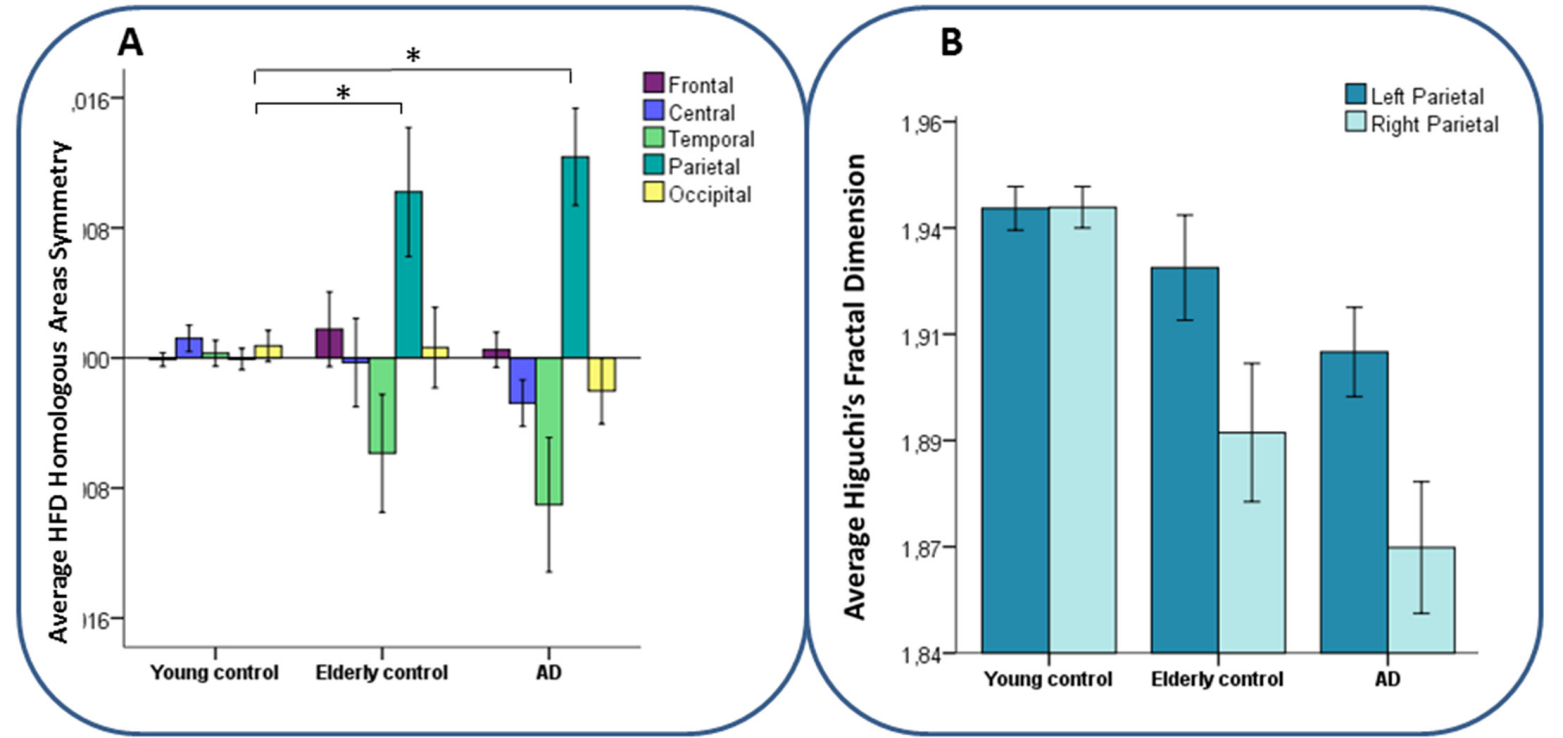
A) Average HFD HArS values of brain regions per group. Deviance from null-line indicates asymmetric HFD in homologous areas. Significant (*p* < .01) group difference in parietal HFD HArS are indicated by a star (*). B) Average HFD values of left and right parietal regions per group. Parietal asymmetry in elderly controls and AD patients is caused by a stronger HFD decline in the right compared to the left hemisphere. Error bars display the 95% confidence interval of the means.

In agreement with the Hemisphere*Group effect in central, temporal and parietal regions when submitting HFD to regional ANOVA models (p < .001 consistently), the asymmetry index of HFD was different among groups in the central, temporal and parietal channel regions (*F*(2,103) = 6.05, *p* = .003; *F*(2,103) = 3.63, *p* = .030; *F*(2,103) = 12.90, *p* < .001 respectively). In detail, the post-hoc comparison in the healthy group showed that YC differed from EC in parietal HArS (*p* = .006), corresponding to symmetric HFD in YC group and left-higher-than-right HFD in elderly subjects (both EC and AD) (Fig 4). Taking together hemispheric values and their inter-hemispheric asymmetries, parietal HFD was reduced bilaterally in elderly subjects, with a stronger HFD decline in the right than in the left region. In line with these results, parietal HFD asymmetry strongly correlated with age in healthy people (Pearson’s *r* = .646, *p* < .001 including YC and EC). Notably, this left-higher-than-right HFD asymmetry is selectively found in the parietal regions, while the other regions show symmetric HFD values in all groups or a tendency towards right HFD dominance in the elderly subjects (Fig 4).

No differences in HFD HArS emerged between EC and AD (*p*>.200 in all regions). In accordance, parietal HFD HArS showed highly similar values in EC and AD (post-hoc difference between groups, *p*>.999). Mirroring the parietal asymmetry similar in healthy elderly subjects and in AD patients, cognitive impairment as assessed by MMSE did not associate with parietal HFD HArS (Pearson’s *r* = -.011, *p* = .918 among elderly subjects, i.e. EC and AD).

## Discussion

Our study, devoted to the whole-brain EEG-channel derived fractal dimension, accomplished four main achievements. First, our results confirmed in an independent sample that fractal dimension depends on age in healthy people and is reduced in AD. In more detail, investigating a complexity measure of brain activity related to scale-free self-similarity of the signal dynamics–the EEG-derived Higuchi’s fractal dimension–we found that HFD increases from adolescence to adulthood and decreases from adulthood to old age. This loss of complexity in neural activity is a feature of the aging brain and worsens with Alzheimer’s disease. Second, we observed a regional prevalence of fractal dimension changes related to aging and AD. The fractal dimension depended most strongly on age in parietal and central brain regions in healthy people, and temporal and occipital fractal dimensions were most strongly related to the impairment in AD. Third, our findings demonstrate that the EEG-derived fractal dimension is reduced with increasing levels of NCC in AD patients. Finally, we found a loss of interhemispheric homologous areas symmetry of parietal fractal dimension that depended on age.

### Fractal Dimension in Healthy Aging

Elderly healthy subjects showed a trend towards whole-brain decline in fractal dimension. We found that over the lifespan of healthy subjects, EEG-derived fractal dimension develops with an inverted U-shape: HFD slightly increases during adulthood until around the age of 60 years, after which HFD significantly decreases with increasing age. These results suggest that the EEG-derived fractal dimension measure is sensitive to a parabolic development of neural efficiency over the life-span. Accordingly, this parabolic maturation of neural capacity over the lifespan is also shown by other brain measures, for example by the inverted U-shaped development of connectivity of cognitive and motor frontal-striatal networks [42], of the optimal balance between local and global neural plasticity [43], of brain weight and of white matter volume [44].

In addition, when considered in dependence on cortical regions, the fractal dimension decreased in elderly controls relative to young controls in central and parietal brain regions, suggesting that fractal dimension decline in healthy aging is partly region-specific. In line with this region-specificity, previous studies showed that large-scale impairments in brain network communication and organization is closely associated with local efficiency declines [44]. Besides, considering that the EEG-derived fractal dimension is typically inversely related to low frequency spectral power of brain activity and positively associated with power in higher frequency bands [12,45], fractal dimension dependence on age seems to mirror the slowing of brain activity that is shown by elderly individuals in central and parietal areas [4,19].

Furthermore, elderly participants displayed a left-higher-than-right fractal dimension asymmetry over the parietal channels. This is in line with findings showing that non-frontal brain regions display less age-related decline in the left hemisphere than in the right hemisphere [18], which has previously specifically been confirmed in parietal areas [19].

### Fractal Dimension in Alzheimer’s Disease

In line with previous findings of declining fractal dimension or other measures of complexity in EEG activity of AD patients [6,7,14,46], we found that HFD is reduced in AD-affected individuals compared to healthy elderly individuals. The size of our sample enhances the reliability of this result. Moreover we found an association between reduced HFD and decreasing cognitive capacity as assessed by MMSE, in line with previous brain activity complexity studies [1,15]. These findings indicate that loss of neural efficiency and reduced cortical communication in AD [44] can be detected with the HFD measure, and provide further evidence strengthening that HFD is sensitive to progressing AD severity.

A plausible partial cause of reduced complexity in brain EEG organization combined with cognitive impairment, might be the reduced capacity in AD patients to compensate for neural damage by means of brain plasticity [43,47]. In favor of this hypothesis, region-specific HFD decline in AD-affected individuals as compared to healthy elderly patients occurred only in temporal and occipital regions. The finding that EEG-derived fractal dimension measured over temporal areas carries AD-relevant information is in line with previous results of HFD reductions and brain activity slowing in temporal regions [1,6].

Notably, we found a significant association between reduced HFD and higher NCC levels. This association showed a trend towards statistical significance when it controlled for the effect of cognitive status (MMSE score), which is known to correlate with NCC levels [22]. Therefore, NCC levels seem to change the EEG-derived fractal dimension. Because elevated levels of NCC in the brain augment oxidative stress and amyloid plaque formation [22], our findings strengthen the notion that increasing levels of NCC occur in parallel with inefficiency of neuronal activity as expressed by the decreased complexity subtending the fractal dimension decline.

Contrary to expectations based on previous findings of reduced connectivity and decreased efficiency in topological organization, especially of frontal brain areas in AD and elderly patients [44,47], we found only moderate and non-significant HFD changes in frontal regions. Our results therefore support that neural changes accompanying aging and AD mainly appear in non-frontal brain regions, in line with brain activity slowing in posterior brain regions [1,3,4].

AD-afflicted participants displayed a parietal left-higher-than-right fractal dimension asymmetry, which did not differ from that of healthy elderly individuals. Consistently, while this parietal interhemispheric asymmetry strongly correlated with age, it did not correlate with cognitive status. The fact that HFD asymmetries found in AD patients were accounted for by only aging and not by cognitive impairment suggests that AD does not create asymmetries in HFD and, hence, affects neural activity complexity bilaterally.

### Diagnostic vs. Read-out Usefulness of EEG-derived Fractal Dimension in Alzheimer’s Disease

Our findings contribute to a host of studies showing that healthy aging is accompanied by similar brain changes as mild stages of AD [3,4,48]. These findings demonstrate the difficulty to find an early neural biomarker to distinguish patients with AD onset from healthy aging individuals. Complexity measures of the EEG signal such as the fractal dimension have been found insufficiently informative of AD to serve as a diagnostic marker [6,7,49]. However, as our and others’ [6,7,14] results show, AD is associated with a sharper decline in fractal dimension of the EEG signal than that of healthy individuals of similar age, which is to some extent region-specific for AD. Thus, measuring the complexity in the EEG signal might contribute to more accurate AD diagnosis and assessment of cognitive abilities. For example, when the fractal dimension is added to a set of other nonlinear measures of the EEG signal as a predictor of AD diagnosis, the diagnosis classification accuracy increases [6,7].

Here, our focus was on specificities of AD-affected vs. healthy elderly, differing changes between healthy elderly vs. young people, with the aim of identifying the regional-specific effects of the disease with respect of healthy aging. Interestingly, a clear regional specificity appeared in the brain complexity modifications. In fact, the brain complexity of elderly people reduced, with respect to the young, in central and parietal regions. Meanwhile, in AD patients we observed reduced activity complexity in temporal and occipital regions. Contrary to our expectations, the inter-hemispheric asymmetry of homologous areas complexity did not differ between healthy and demented elderly people, indicating that AD affects the two hemispheres similarly. This emphasizes the finding that homologous areas asymmetries increase with age, both in healthy people [19] and in those affected by AD. In contrast with the regional specific complexity alterations in AD vs. healthy aging in temporo-occipital cortices, we found that the brain complexity reduction that best correlated with NCC levels in AD patients was in the parietal region. These regional specificities in brain complexity suggest that the HFD of particular cortices can provide a readout measure of specific aspects related to the disease (such as NCC levels). It can be useful to monitor brain activity-modifying interventions as, for example, has been done using the 3D fractal dimension of segmented lung vessels in monitoring pulmonary hypertension [50,51].

### Limitations

Although we argue that our results support the sensitivity of the EEG-derived HFD measure for neural changes in healthy and pathological aging, our study faced some limitations. First, we calculated HFD from EEG channel signals. Each EEG-channel’s time series represents a signal that is a mix of activities from multiple brain sources. Thus, the channel signal does not only represent activity from the region lying directly underneath, but even sufficiently strong, distant currents induce signal in the recording location. Investigating the EEG data with more sensitive region-specificity requires separating the cortical source contribution, as done for example by LORETA procedures [3,4]. However, while these algorithms provide the power distribution of the oscillatory rhythms of the involved cortical regions, they do not provide temporal dynamics. Thus, we calculated the fractal dimension directly from the time series obtained from the EEG channel recordings which is, to our knowledge, in accordance with all previous studies. Despite the imprecision of channel-derived region-specificity with respect to brain sources analysis, our data indicate that there are, to some extent, region-specific behaviours of the fractal dimension in healthy aging and in AD. Another approach to source reconstruction, which in contrast to LORETA provides the identified source time behaviour, is represented by the Functional Source Separation algorithm (FSS) [52,53]. We did not use FSS in the present study, since up until now it has been effective only for primary cortical districts under specific experimental settings.

Second, here we present data from EEG recordings with low sampling frequency and a relatively small number of electrodes. Modern EEG recordings with a higher sampling frequency might optimize the HFD measure, and higher electrode density could be beneficial in investigating further region-specific HFD fluctuations. Comparative analyses with other current analysis procedures will reveal whether the fractal dimension of the EEG signal provides a more sensitive index than others to age-related neural changes or the quality of aging in healthy people. Moreover, further studies will evaluate whether or not EEG-derived HFD can be useful in early AD diagnosis or monitoring AD in patients with elevated levels of NCC [22].

Finally, we found that there is a discrepancy in HFD values and range of HFD values between different studies, despite the use of the same algorithm and similar EEG parameters (19 channels, resting-state, eyes closed). For example, the values following from our HFD calculation are higher than the values found in another AD study [6] and are higher than the values observed in previous studies on stroke and lifespan neural development that were done in collaboration with our group [12,25]. These latter studies also reported HFD values in a much wider range (HFD values of approximately 1.25–1.60), in comparison to our results. In our data, we examined dependence of HFD values on sample frequency and *k*_*max*_, but we found that changing these two parameters did not affect the HFD values substantially (Fig 2). One plausible cause of variability in HFD values might be the different references used to re-reference EEG signals; where we used an average reference, our collaborators [12,25] used a reference at a point of infinity. Future studies should aim to discover how EEG reference influences HFD values, or what other factors may cause significant variability in HFD values, in order to make studies of EEG-derived fractal dimension more comparable and to possibly facilitate translation to the clinical application of the HFD measure.

## Conclusions

We found that the EEG-derived Higuchi’s Fractal Dimension measure is sensitive to neural changes selectively related to healthy aging and to Alzheimer’s Disease. Specifically, we found that HFD is sensitive to the maturation of brain function over the life span and to disease progression in AD. Furthermore, our results show that HFD to some extent captures region-specific neural changes in healthy aging and in AD.

## Supporting Information

S1 Dataset Dataset including all subjects’ age, HFD values per channel, and MMSE scores and NCC values if available. (XLSX)
